# Decal bone matrix as a local antibiotic delivery vehicle in a MRSA-infected bone model: An experimental study

**DOI:** 10.4103/0019-5413.65140

**Published:** 2010

**Authors:** Shyam K Saraf, Awadhesh Yadav, Santosh Nagwani, MR Sen

**Affiliations:** Department of Orthopaedics; 1 Medicinal Chemistry, Institute of Medical Sciences, Banaras Hindu University, Varanasi, Uttar Pradesh, India; 2 Microbiology, Institute of Medical Sciences, Banaras Hindu University, Varanasi, Uttar Pradesh, India

**Keywords:** Allograft, decal bone matrix, osteomyelitis, local delivery system, vancomycin

## Abstract

**Background:**

Polymethyl methacrylate (PMMA) antibiotic beads though have proved their utility as a local antibiotic delivery system, however, there are limitations. Decalcified bone matrix (DBM) as a vehicle of antibiotics can serve the purpose, provided a minimum inhibitory concentration is sustained. Healing of the defect and avoiding the second surgery is another advantage. We studied the DBM as the delivery vehicle for vancomycin in controlling the methicillin-resistant *Staphylococcus aureus* (MRSA) osteomyelitis as well as healing of the cavity simultaneously in an experimental study.

**Materials and Methods:**

An *in vitro* study was conducted to optimize vancomycin impregnation in the DBM. For the *in vivo* study, a unicortical defect was created in the metaphysis of the distal femur in 18 rabbits. After contaminating the defect with MRSA, rabbits were divided into three groups. Group I (eight limbs) received no graft. Defects in group II (11 limbs) were filled with plain DBM chips and in group III (14 limbs), cavities were implanted with vancomycin-impregnated decal bone chips. Rabbits were assessed by clinical, radiological, histological, gross examination and bacterial load assay. High Performance Liquid Chromatography HPLC analysis of vancomycin in group III was done to assess the concentration in DBM chips.

**Results:**

In group I, the infection persisted throughout the period of the study. Group II showed the fulminated infection at the grafted site with DBM chips sequestrating out. Vancomycin-impregnated decal chips in group III did not show any sign of infection and eventually incorporated. The bacterial load study showed a progressive load change and HPLC revealed an effective antibiotic concentration up to 3 weeks in both *in vitro* and *in vivo*.

**Conclusion:**

Decal bone chips were effective as the local antibiotic delivery vehicle in preventing the MRSA osteomyelitis model. It eluted vancomycin significantly and the graft uptake was also excellent. Allogeneic decal grafts eliminated the need for second surgery and acted as an excellent delivery vehicle for antibiotics.

## INTRODUCTION

The treatment of bone and joint infections and resulting bony defects is a challenge to the orthopedic surgeons as there are two major problems to overcome: control of infection and filling the bony defect. A systemic antibiotic may not be effective to control the infection because of poor penetration into the bone.^1^ The treatment becomes more difficult and complex with the emergence of highly resistant pathogens such as methicillin-resistant *Staphylococcus aureus* (MRSA).

The advantage of local delivery system of antibiotics is that it provides a high drug concentration at the site of infection without systemic side effects. It can penetrate avascular areas of wound and the bone and is effective against the sessile bacteria in a biofilm.^2^ The most comprehensive information regarding local drug delivery has been provided by combination of polymethyl-methacrylate (PMMA) with gentamicin which has been used extensively in the past.^3^-^5^ The use of PMMA as a drug delivery vehicle has few disadvantages like need for a second surgery for the removal of beads or limited choice of antibiotics which can be combined with PMMA as not all the antibiotics are heat stable.^6^ Further, gentamicin is not the agent of choice for infections with MRSA. After complete elution of the antibiotic, PMMA may even act as a foreign body for the growth of surviving bacteria.^7^

An ideal antibiotic vehicle should be able to carry an antibiotic having a bactericidal activity against most of causative pathogens including MRSA. It should be able to provide a sustained high concentration at the site of infection without local or systemic toxicity. In addition, whenever necessary, it should help in healing of bony defects without further surgery. Allogeneic decal bone was chosen as it has been proved to be effective in healing of bony cavities and bone defects.^8^-^10^ Vancomycin-impregnated bone grafts have been used for filling infected bone defects and nonunions, and in infected joint replacement procedures.^11^-^13^ We selected vancomycin as the antibiotic to be impregnated as it has shown to be effective against both coagulase-positive and -negative MRSA.^14^ The studies of antibiotic-impregnated decalcified bone matrix (DBM) in the literature are scarce;^15^ hence the present experimental study aims at evaluating the DBM chips as a local antibiotic delivery vehicle for vancomycin in MRSA-infected osteomyelitis cavities.

## MATERIALS AND METHODS

The present experimental study was started only after approval from the animal ethical committee of our institute. Mature healthy rabbits of either sex weighing between 1.5 and 2.5 kg were chosen as subjects. In all the rabbits, a defect of approximately 6 mm was created at the distal femoral metaphysis. It was infected with a measured aliquot of MRSA strain. The rabbits were then divided in three groups: group I, where the defect was left as such without graft material; group II, where the defect was grafted with plain DBM chips; and group III, where the defect was grafted with vancomycin-impregnated DBM chips. The study was carried out in the following steps.

### Preparation of DBM chips

A mature healthy rabbit was sacrificed with a lethal dose of sodium pentobarbital intravenously. Under strict asepsis, long bones were stripped off their attached soft tissues and were put into 0.6 N HCl at room temperature for 4–6 h for decalcification. The completion of process of decalcification was judged by the soft leathery texture of the matrix and its translucency. The explant with the completion of demineralization started floating at the surface of the solution. The DBM thus obtained was washed thoroughly with sterile distilled water and cut into about 3-mm size pieces. The bone chips thus prepared were kept in 70–90% ethanol at 2°C for minimum of 48 hours for further use.

### Antibiotic impregnation of DBM chips

Vancomycin powder obtained from AstraZeneca Pharma India Ltd was dissolved in distilled water (1 g/10 ml). The prepared DBM chips after taking out from ethanol were washed thoroughly with distilled water and then were incubated in this known concentration solution for 24 h at 37°C to be used as a graft in group III. Prior to the *in vivo* study, an *in vitro* concentration analysis of vancomycin-impregnated DBM chips was done by the High Performance Liquid Chromatography HPLC system consisting of a LC-10-AT pump (Shimadzu, Kyoto, Japan), a SIL-10A auto-injector (Shimadzu, Kyoto, Japan), and a SPD-10A UV-VIS detector (Shimadzu, Kyoto, Japan) with a phenomenex (USA) RPC_18_ column (250 mm × 4.6 mm × 5 μm) protected by a guard column (LC18). The concentration of vancomycin in the sample was determined as per method of Raju *et al*.^16^

### Operative technique

**Figure 1:**
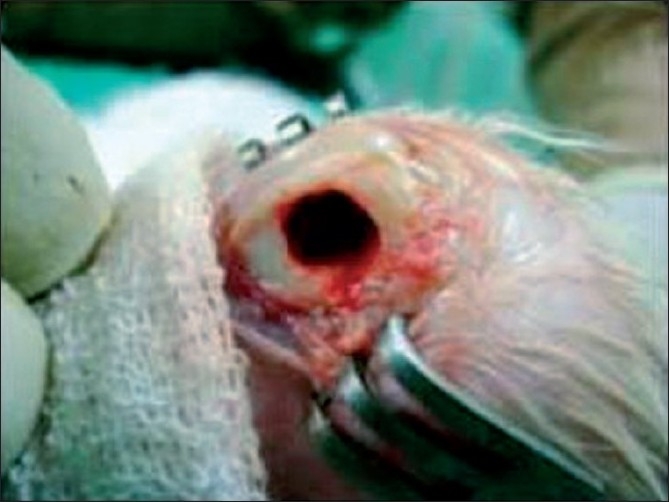
Intraoperative photograph showing 6 mm unicortical metaphyseal defect in distal femur

The rabbits were anesthetized using 2 mg/kg of ketamine hydrochloride and 2 mg/kg of midazolam (Sedoz, Claris Lifesciences Ltd). Both the lower limbs were operated one by one in a single sitting under standard aseptic precautions. A 2-cm-long incision was given at the lateral aspect of distal end of the femur and the metaphyseal region was exposed. With the help of a hand drill, a 6-mm-diameter unicortical defect was created [Figure 1]. Post operatively no antibiotics were given.

### Induction of infection

A known strain of MRSA obtained from a patient of chronic osteomyelitis with known antibiogram was used to prepare a homogenous suspension in normal saline. The approximate bacterial concentration of the suspension was determined by comparing with 10^6^concentration Brown’s opacity tube.^17^ A 10 μl suspension of the MRSA strain containing 5.5 × 10^6^ colony forming units was then instilled into the metaphyseal defect.

We started our *in vivo* study with the group I rabbits first where no graft material was used. This was to establish the osteomyelitis model before undertaking the studied on group II and group III rabbits. The wound was closed with absorbable sutures. The limbs were not splinted in any of the groups and the rabbits were left free in the cage. In group II and group III rabbits, the infected metaphyseal defect was immediately filled with a uniform size of plain DBM chips and antibiotic-impregnated DBM chips, respectively.

The results were assessed by clinical, roentgenographic, bacterial load assay, gross and histopathological examination. The incision site was examined for signs of infection, namely, induration, edema, pus discharge, abscess formation, gaping, and the position of the graft. The general condition of the animal, weight bearing on the operated limbs, loss of hair, and loss of weight were observed. Periodic X-rays of both lower limbs were taken on postoperative day 1 and then at weekly intervals till the animals were sacrificed as per schedule [Table 1]. The observations were made regarding radiodensity, margins of the graft and the walls of cavity, status of the host graft union and complications like pathological fracture and extrusion of the graft. Pus discharge from group I and group II rabbits was sent for culture to rule out any opportunistic infections. Biopsy specimens obtained from the walls of the defect in all the three groups of the rabbits sacrificed at 2, 4, and 6 weeks were assessed for bacterial load assay. Gross examination of the freshly dissected specimen was done to study evidence of infection like pus discharge, infected granulation tissue, sequestrum. The color, surface, consistency of the bone chips, evidence of incorporation of the graft with host bone and any complication like pathological fracture, extrusion of graft were observed. For histopathological examination, the cut sections through the grafted site were prepared by the standard method, stained with hematoxyline and eosin and examined under light microscope for the status of osteomyelitis and fate of DBM chips.

**Table 1:** Details of the experimental design

Group	No. of limbs	Duration for observation						
		24 h	3 d	1 wk.	2 wk.	3 wk.	4 wk.	6 wk.
I(NCG)	8	–	–	1	2	2	3	–
II(PCG)	11	–		1	2	2	2	4
III (EG)	14	2	2	2	2	2	2	2

h = Hours; d = Day; wk. = Week, NCG = Negative control group, PCG = Positive control group, EG = Experimental group

For HPLC analysis of the local antibiotic concentration in the grafted region, immediately after sacrificing the animal (group III rabbits), the tissue samples were obtained from the grafted site at 24 h, 3 days, 1 week, 2 weeks, 3 weeks, and 4 weeks when animals were sacrificed. These samples were pretreated with 30% TCA (trichloroacetic acid) to precipitate proteins. The mixture was centrifuged at 12,000 rpm for 15 min and the 20 μl supernatant was injected into the HPLC column. Results were calculated directly by peak area comparison with the vancomycin standard calibration curve.

### Observation

#### In vitro study observations

The elution study of antibiotics showed a logarithmic decrease of the concentration over the period of 4 weeks. The highest concentration was on day 1 (60±2.86 μg/l00μl) decreasing to 2±0.29 μg/ l00 μl on day 28. The study shows that the concentration of vancomycin in the sample was above the MIC level 1 to 4 μg/ml^18^ for MRSA pathogens.

#### In vivo study observations

A total of 33 limbs were included in the study. The animals were kept in standard steel cages, one per cage. They had free access to water and feed (animal food pallets and vegetables) and were observed daily by an animal care taker and our team members. The rabbits operated were divided into three groups; Group I (eight limbs), group II (11 limbs) and group III (14 limbs) [Table 1].

### Clinical observations

**Figure 2:**
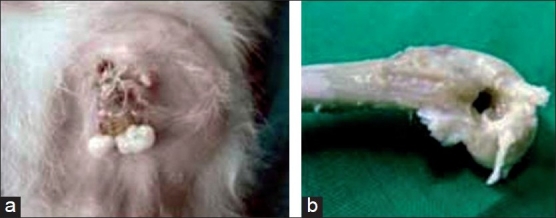
Group I rabbit: (a) a clinical photograph showing edema and induration with pus discharge at day 7 and (b) gross examination at week 4 showing the patent metaphyseal defect with infected tissues

**Figure 3:**
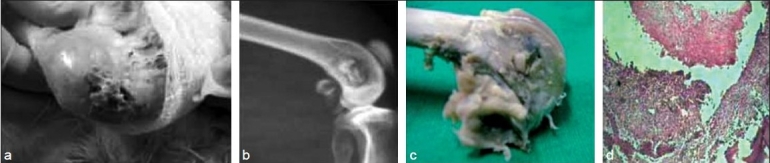
Group II rabbit: (a) a clinical photograph showing a protruding decal chip at the second week; (b) Roentgenogram showing sequestrated DBM at 6 week; (c) gross examination at 2 week showing the grafted decal bone; (d) photomicrograph showing evidence of infection with extensive neutrophilic exudates and necrotic material at the second week (Hematoxylin and eosin, ×40)

**Figure 4:**
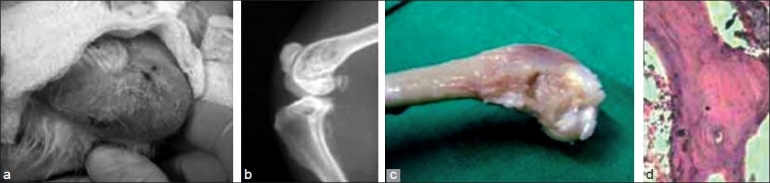
Group III: (a) a dry suture line at the third week; no graft protrusion; (b) roentgenogram and (c) autopsy showing incorporation of the graft at 6 weeks; (d) a photomicrograph showing the incorporation of the graft and woven bone formation with marrow components at 6 weeks (Hematoxylin and eosin, ×100)

In group I and II, poor general condition with evident pus discharge was observed in all the rabbits. The discharge started from the suture line as early as on day 3. Edema, erythema, and induration were seen in both the groups at 1 week [Figure 2a]. The presence of a thick, poultice-like material was also observed in 7 limbs in group II. In six limbs, DBM chips were extruded out from the suture line with evident pus at 2 weeks [Figure 3a]. In group III rabbits, two limbs showed serous discharge from the suture at day 3. It subsided at 1 week and the wound healed normally. In all other limbs, the suture line was healthy and dry at day 7. There were no signs of infection from the suture line in any of the operated limbs [Figure 4a].

### Roentgenographic observations

In group I rabbits, soft tissue swelling at the operative site suggested pus collection. The defect created at the distal femur showed clear margins. At 4 and 6 weeks, the metaphyseal defect was seen as such; however, the tissue edema had subsided. In group II, changes typical of osteomyelitis such as osteolysis, sclerosis, and sequestrum formation were visible. No signs of incorporation like graft becoming irregular, fluffy and more radiodense, margins of the defect becoming hazy, were seen. No signs of host graft union were observed [Figure 3b]. In six limbs, graft appeared to be protruding out starting as early as 3 weeks. In group III, no signs of infection like increased soft tissue swelling, osteolysis, sclerosis, or protrusion or sequestrum were seen. All impregnated grafts showed signs of incorporation at 6 weeks [Figure 4b].

### Bacterial load assay

The infection persisted up to 4 weeks in all rabbits of group I and group II [Table 2]. Biopsy specimens of group III rabbits at week 2, 4, and 6, respectively, showed no organism/growth.

**Table 2:** Mean bacterial count of the MRSA strain in various groups

Duration	GroupI	Group II	Group III
Day 7	3.5 × 10^6^ CFU/g	2.0 × 10^6^ CFU/g	2.2 × 10^2^ CFU/g
Week 2	1.6 × 10^6^ CFU/g	1.5 × 10^6^ CFU/g	0
Week 4	1.4 × 10^6^ CFU/g	1.2 × 10^5^ CFU/g	0
Week 6	0	0	0

### Gross examination at autopsy

In group I, thick pus collected around the metaphyseal defect in all the rabbits in the second week. The defect was filled with necrosed and fibrous tissues [Figure 2b]. Group II rabbits also showed collection of thick pus around the operated site with the graft lying as such without any signs of incorporation. In six, the plain bone graft was protruding outside the suture line [Figure 3c]. Group III rabbits showed no pus collection or any sign of persistent infection at the operated site and grafts fully incorporating at 6 weeks [Figure 4c].

### Histological examination

Group All rabbits of Group I showed evidence of pyogenic infection in the form of an extensive neutrophilic exudate and necrosis without any evidence of new bone formation, though the amount of necrosis had decreased at 4 weeks. Group II rabbits showed plenty of neutrophilic exudates, bundles of collagen with interspersed fibrous tissue cast surrounding the grafted DBM chips. No histological evidence of new bone formation or graft incorporation was observed [Figure 3d]. Group III rabbits at 2 weeks showed new bone formation with osteocytes and laying down of matrix with minimum inflammatory exudates. At 4 weeks, gradual incorporation of the grafted material with irregular fibrous strands laying in the canal was observed. Around 6 weeks in this group, the grafted material was incorporated with evidence of woven bone along with marrow components [Figure 4d].

### High performance liquid chromatography analysis

The mean concentration of vancomycin over a period from day 1 to week 4 was calculated using the analysis software with the help of standard calibration curve. The highest initial concentration of vancomycin measured at day 1 (60±2.5 μg/100 *μ*l) decreased to 1±0.45 *μ*g/100 *μ*l at the end of 4 weeks [Table 3].

**Table 3:** Result of the *in vivo* study of the vancomycin concentration at the grafted site

Duration	Mean area under curve by HPLC ± SD	Mean concentration of vancomycin
Day 1	417261±12720	60 ±2.5mg / 100 ml
Day 3	203967±13937	27±3.8 mg / 100 ml
Week 1	130736±7558	15± 1.9mg / 100 ml
Week 2	50140±3174	7± 5.6mg / 100 ml
Week 3	25659±2174	3± 4.1mg / 100 ml
Week 4	8534±316	1 ±0.45mg / 100 ml

## DISCUSSION

The local antibiotic delivery system seems to be a useful and safe method of treating the complex problems of open fractures and osteomyelitis, by maximizing the local concentration of antibiotics while minimizing their systemic toxicity.^19^ In avascular bones, it may not be possible for systemic antibiotics to achieve local therapeutic concentrations.^20^ Once microorganisms attach to bone or other biomaterials, the bacteria become metabolically less active and concomitantly cover themselves with a biofilm, rendering them unresponsive to usually therapeutic antibiotic levels. To be effective against bacteria in a biofilm, the desired antibiotic concentrations must be 10–100 times the usual bactericidal concentration.^20^ Giving the antibiotic by parenteral route or locally without any local drug delivery vehicle may not be beneficial due to the unpredictable antibiotic concentration.^2^ In our study, we chose the MRSA strain to create the osteomyelitis model as MRSA infections are even more difficult to manage. Vancomycin was selected as the drug of choice for the reason that it is effective against most gram-positive pathogens and infections with MRSA.^14^

In 1970, Bucholz introduced the mixing of antibiotic with palacos resins^21^ and since then there have been several clinical studies on antibiotic-loaded bone cement.^22^,^23^ Despite the widespread use of PMMA in local antibiotic therapy, it has limitations like need for second surgery for removal of beads. The choice of antibiotic used with PMMA is limited as not all the antibiotics are heat stable. The biodegradable alternatives to bone cement to provide a high, effective concentration of antibiotics at the site of the wound are current research topics. The use of bone grafts as a carrier for antibiotics has been described by many authors.^12^,^24^,^25^ Since an autologous bone is not always readily available in clinical practice, in our experimental study we used the allogeneic decal bone. The decalcified bone graft has shown to be an osteoinductive and osteoconductive material under various experimental conditions. The insignificant immunological reaction refutes the concerns about the antigenicity.^10^,^26^,^27^ Winkler *et al*.^28^ investigated the combined effects of compounds of bone graft and antibiotics on bone repair and eradication of infection, with both gram-positive and -negative pathogens. The concentration of antibiotics in the albumin was well above the MIC for common pathogens throughout the investigation in all tested specimens. Similar results of elution studies were observed in our *in vivo* study using antibiotic-impregnated DBM chips. Edin *et al*.^29^ investigated the adverse effects of antibiotics over bone regeneration and fracture healing and found that local levels of vancomycin of less than 1000 μg/ml had little or no effect on osteoblast replication but concentrations of 10,000 μg/mL caused cell death.

In our study, group I rabbits served as control and developed MRSA pyogenic osteomyelitis in all the limbs. The mean bacterial count was 3.5 × 10^6^CFU/g at 1 week decreasing to 1.4 g ×10^6^CFU/g at 4 weeks suggesting the persistent infection at the operated site. Group II rabbits showed no signs of incorporation of grafted DBM chips in any of operated limbs; rather six limbs of group II rabbits had shown protrusion of grafted DBM chips. The mean bacterial count was 2 × 10^6^CFU/g at day 7 falling to 1.2 × 10^5^ CFU/g at the end of week 4. All limbs of group III rabbits showed complete incorporation and the mean bacterial load was zero. The elution study of antibiotic showed the vancomycin concentration well above the MIC for the MRSA strain (2 μg/100 ml) up to 3 weeks.

DBM chips used in our study as a local antibiotic delivery vehicle worked as an excellent carrier for vancomycin over a period of 3 weeks for the treatment of MRSA strain infection. DBM chips were completely incorporated and new bone formation was observed in all grafted rabbits without any signs of infection. Hence the antibiotic-impregnated decal bone graft can accomplish the eradication of infection and grafting of bony defects as a single-stage procedure.
